# The impact of surgery-first approach on the oral health-related quality of life: a systematic review and meta-analysis

**DOI:** 10.1186/s12903-019-0842-1

**Published:** 2019-07-08

**Authors:** Xinqi Huang, Xiao Cen, Wentian Sun, Kai Xia, Liyuan Yu, Jun Liu, Zhihe Zhao

**Affiliations:** 1 State Key Laboratory of Oral Diseases & National Clinical Research Center for Oral Diseases, West China Hospital of Stomatology, Sichuan University, No. 14, 3rd Section, South Renmin Road, Chengdu, 610041 Sichuan China; 2 Department of Orthodontics, West China Hospital of Stomatology, Sichuan University, Chengdu, 610041 People’s Republic of China; 3 Department of Temporomandibular joint, West China Hospital of Stomatology, Sichuan University, Chengdu, 610041 People’s Republic of China

**Keywords:** Surgery-first approach, Dentofacial deformity, Oral health-related quality of life, Systematic review, Meta-analysis

## Abstract

**Background:**

The oral health-related quality of life (OHRQoL) is affected by dentofacial deformity. Patients with dentofacial deformity are normally treated with orthognathic surgery, including conventional three-stage method (CTM) and surgery first approach (SFA). The aim of this systematic review and meta-analysis was to compare the impact of SFA with CTM on the OHRQoL of patients with severe dentofacial deformity.

**Methods:**

Five English databases, three Chinese databases, and six grey literature databases were searched (January 2000 to July 2018). Randomized controlled trials, controlled clinical trials, and cohort studies assessing the OHRQoL of patients who underwent SFA or CTM were included. After selecting studies, extracting data, and assessing risk-of-bias according to the Cochrane Handbook for Systematic Reviews of Interventions and the Newcastle-Ottawa Scale, meta-analysis was performed to elucidate the effects of SFA on the changes of OHRQoL of patients with dentofacial deformity at each stage and made a comparison with CTM.

**Results:**

There were 4 studies with 122 participants were selected for the final analysis. Three among these studies were included in meta-analysis, 2 of which were included in each forest plot. All the included studies were graded as moderate value of evidence according to GRADE quality analysis. Over the period of 2-year follow-up after bonding, the OHRQoL of the patients in SFA group showed an improving trend and was better than those in CTM group generally. After debonding, the summary scores of the 14-item Oral Health Impact Profile (OHIP-14) (− 2.92, *P* = 0.12) and Orthognathic Quality of Life Questionnaire (OQLQ) (− 5.59, *P* = 0.01) were smaller in SFA group than CTM group.

**Conclusions:**

Clinical evidence indicates that SFA can contribute to the better OHRQoL in patients with dentofacial deformity immediately and persistently.

## Background

Oral health-related quality of life (OHRQoL) is a multidimensional construct reflecting the comfort of eating, sleeping, and engaging in social interaction, which measures the impact of oral health on emotion, society, and function of daily life [1]. Gender has shown to influence OHRQoL [2, 3]. It is also reported that disadvantaged adolescents might experience poor OHRQoL due to the worse oral condition, which suggested socioeconomic position was an important factor of OHRQoL [4].

Dentofacial disharmonies are observed in 38.5% of adolescents [5, 6], which severely affect daily life such as breathing, speaking, and eating. These patients are also potentially with self-abasement and socially awkward, which afflicts psychosocial development [7–9]. Therefore, dentofacial deformities have seriously unfavorable influence on the patients’ OHRQoL [10].

The conventional orthognathic surgery, also named as conventional three-stage method (CTM), generally consists of presurgical orthodontic treatment, surgery treatment, and postoperative orthodontic adjustment [11]. According to this protocol, presurgical orthodontic treatment, which is essential to the final stable surgical results, will focus on removing dental decompensation, aligning and leveling the teeth, and coordinating dental arches [12]. This process, however, can hardly contribute to the changes of vertical and transverse plane [13]. It was indicated that the correction of transverse discrepancy could be addressed with surgery without presurgical orthodontic treatment [14]. In addition, there are gingival recession, enamel decalcification, root resorption, and worsening lip profile during presurgical orthodontic treatment [15, 16]. These results inevitably have a negative effect on the aesthetic and psychologic aspects. It was reported that the OHRQoL of patients in the presurgical orthodontic treatment was worse than that in the postsurgical treatment phase [17].

In 1988, Behrman raised the concept of “surgery first and orthodontics second”, which was also named as surgery first approach (SFA) [18]. They hypothesized that once the jaw position was counterbalanced, the normalized soft tissues would facilitate the postoperative tooth movement, and thus shorten the duration of treatment. Clinical data from Chang Gung University also demonstrated that the surgical environment might bring about 4-month period of higher bone metabolic changes and osteoclastic activities, possibly accelerating postoperative tooth movement [19]. It was indicated that postoperative alignment was similar to that in Class I cases, and any possible surgical relapse could be rectified with postoperative orthodontics [20]. Based on this approach, a preplanned orthodontic treatment is fulfilled after the surgery, yielding promotion in the facial aesthetics straight away and providing a decreased total treatment time [21, 22]. These components may lead to the enhancement of patients’ cooperation and better OHRQoL [23, 24]. A large curve of Spee, however, may hamper the accurate evaluation of a predictable mandible position and final occlusion, resulting in difficulties in designing treating plan [21]. It was also exhibited that the relapse rate of SFA group was higher than that of CTM [25, 26].

Previous systematic reviews mainly focused on the effectiveness of SFA [13, 27–30]. Only one of them suggested that the immediate improvement in facial esthetics might have a positive influence on OHRQoL roughly, which only described two studies on this aspect and did not involve the relevant results of different periods or perform the quantitative synthesis [29].

In this systematic review and meta-analysis, we searched the literatures, evaluated the quality of clinical trials, and summed up the evidence to elucidate the effects of SFA on the changes of OHRQoL of patients with dentofacial deformity at each stage and made a comparison with CTM.

## Methods

### Protocol and registration

This systematic review protocol was registered under the PROSPERO register with the number CRD42018099063 (www.crd.york.ac.uk/prospero).

### Eligibility criteria

Only full-length articles, which satisfied following criteria based on PICOS schema, were considered for inclusion in this systematic review.

1. Patients (P): all participants, with no restriction on age, gender, or malocclusion types.
2. The intervention group (I): SFA.
3. The control group (C): CTM.
4. Types of outcome measurements (O): OHRQoL, with no filter for the instruments.
5. Study types (S): prospective and retrospective studies including randomized clinical trials (RCTs), controlled clinical trials (CCTs), and cohort studies.

### Exclusion criteria

1. Intervention without orthognathic surgery.
2. Outcomes without data on OHRQoL.
3. Repetitive publication.

### Information sources and search strategy

Two calibrated reviewers (X.H. and X.C.) conducted the electronic searches independently, and any disagreements were solved by discussion or judged by the third reviewer (J.L.).

Five English databases (i.e. Cochrane Database of Systematic Reviews, the Cochrane Central Register of Controlled Trials, PubMed, Embase, and Medline (via Ovid)), three Chinese databases (i.e. China National Knowledge Infrastructure, Chinese Biomedical Literature Database, and VIP Database), and six grey literature databases (EOS abstract index, IADR abstract index, clinicaltrials.gov, ISRCTN registry, Grey Literature Report, and Open Grey) were searched from January 2000 to July 2018 (updated to July 2018), with no language restrictions. We also checked the reference lists of the included studies to guarantee the inclusion of all relevant studies. The details of the Medline (via Ovid) search were shown in Table 1.

**Table 1:** Details of Medline (via Ovid) search

1. exp. “Quality of Life”/	
2. QoL.mp.	
3. OHRQoL.mp.	
4. oral health-related quality of life.mp.	
5. 1 or 2 or 3 or 4	
6. Orthognathic Surgery/	
7. surgery first approach.mp.	
8. exp. Orthognathic Surgical Procedures/	
9. surgery first.mp.	
10. 6 or 7 or 8 or 9	
11. 5 and 10	

### Selection of studies

Two calibrated reviewers (X.H. and X.C.) checked the titles and abstracts of the identified records and screened out obviously irrelevant ones, and then evaluated full text reports for potentially suitable studies, independently and in duplicate.

If supplemental information was required, these two reviewers would contact the corresponding author of the study and this study would be categorized as awaiting assessment. Any disagreement was judged by two adjudicating senior authors (J.L. and Z.Z).

### Data extraction

Two of the authors (W.S and K.X) extracted and recorded the data independently. Two adjudicating senior authors (J.L. and Z.Z) resolved the referred disagreement.

The source and nationality of participants, treatment types, follow-up periods, and outcome measurements were recorded. The participants information also included gender, number, and malocclusion types. The follow-up periods were classified into after bonding, after surgery, and debonding. The outcomes were the scores of OHRQoL, which could be measured by different kinds of questionnaires, and information of the instruments was also extracted.

### Quality assessment

Two reviewers (X.H. and X.C.) were calibrated and independently assessed the risk of bias of the included randomized trials by the Cochrane Handbook for Systematic Reviews of Interventions from six separate domains: random sequence generation; allocation concealment; blinding of participants and personnel; blinding of outcome assessment; incomplete outcome data; selective reporting [31].

For the nonrandomized studies, we judged the risk of bias with the Newcastle-Ottawa Scale and employed “star system”. One study could be awarded one star for each numbered item when it fulfilled certain criteria, which was regarded as low risk of bias. When there was no description in the study, the risk of bias was regarded as unclear, while the other conditions were regarded as high risk of bias [32]. (Table 2).

**Table 2:** Items and criteria of the Newcastle-Ottawa scale for quality assessment

	Items	When to give stars (low risk of bias)
Selection	Representativeness of the exposed cohort	truly or somewhat representative of the average in the community
	Selection of the control group	Drawn from the same community as the exposed cohort
	Ascertainment of the treatment group	Secure record or structured interview
	Demonstration that outcome of interest was not present at start of study	Yes
Comparability	Comparability of participants on the basis of the design or analysis	Study controls for the most important factor or any additional factor
Outcome	Assessment of outcome	Independent blind assessment or record linkage
	Was follow-up long enough for outcomes to occur?	Yes
	Adequacy of follow-up	Complete follow-up, or Subjects lost to follow-up unlikely to introduce bias, or small number lost follow-up, or description provided of those lost

Each study was graded with A, B, or C, based on the GRADE quality analysis criteria [33]. (Table 3).

**Table 3:** Criteria of GRADE quality analysis

Definitive grade	Criteria
Grade A (high value)	Randomized clinical study or a prospective study with a well-defined control group; Clear definition of diagnosis and endpoints; Description of diagnostic reliability tests and reproducibility tests; Blinding of outcome assessment.
Grade B (moderate value)	Cohort study or retrospective study with a well-defined control group; Clear definition of diagnosis and endpoints; Description of diagnostic reliability tests and reproducibility tests.
Grade C (low value)	Large attrition; Unclear definition of diagnosis and endpoints; Ill-defined patient material.

### Meta-analysis and sensitive analysis

It is nonsensical to combine the comparisons of different treatments with different comparators, or combine the diverse outcome measurements [34]. Therefore, a meta-analysis was planned to perform, when the comparisons of treatments and follow-ups were similar, and the instruments to measure OHRQoL were same.

For continuous data, mean difference and 95% confidence intervals (CIs) would be used. The measurements of SFA effect for binary data were to be shown as relative risks along with 95% CIs. The statistical significance was set at α = 0.05 (two tailed z tests) [35].

Statistical heterogeneity was tested by the chi square and I^2^ tests [36]. If heterogeneity was high (I^2^>50%), the random-effects model was selected for the meta-analysis. Otherwise, the fixed-effects model was chosen [36].

Sensitivity analysis would be conducted to explore the possible source of heterogeneity. The “leave one out” approach, when more than two studies were included, was planned to evaluate the effect of each study on the summary risk.

## Results

### Study selection

**Fig. 1:**
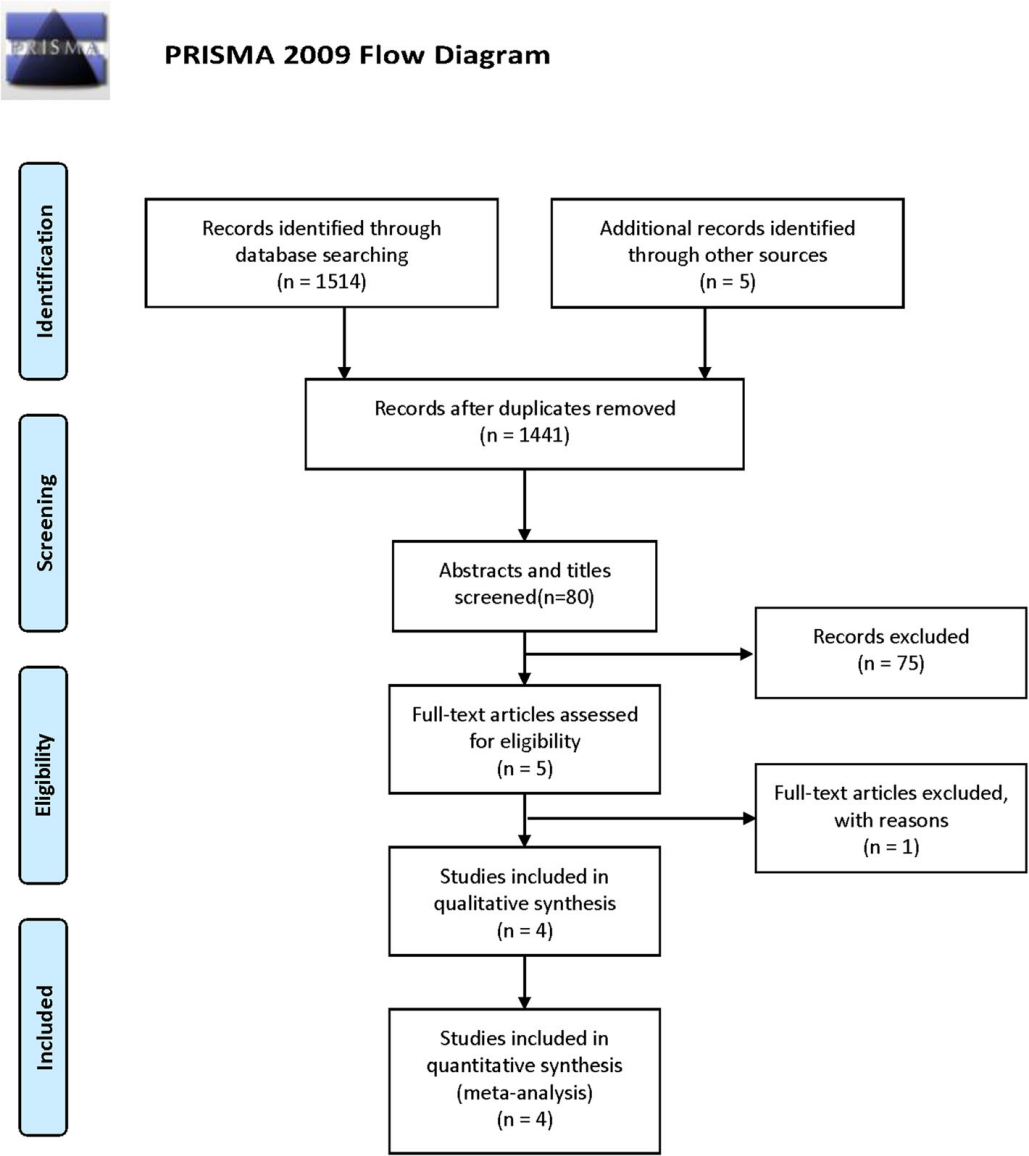
Flow of information through the different phases of the systematic review

Four studies, including 122 participants (59 subjects for SFA and 63 subjects for CTM) published between 2015 and 2017, were included in the final analysis [37–40]. The references of these studies were examined, but yielded no studies for evaluation. The flow diagram is shown in Fig. 1.

### Study characteristics

The characteristics of eligible studies are presented in Table 4. One of included study was retrospective non-RCT [37], while two studies were prospective non-RCTs [38, 39] and the other one study was prospective RCT [40]. Three studies enrolled skeletal Class III patients [37–39], while the other one studied the patients with skeletal Class III and II malocclusion [40]. All of them were treated with SFA or CTM. One study employed Orthognathic Quality of Life Questionnaire (OQLQ) [37], while another one study applied 14-item Oral Health Impact Profile (OHIP-14) [38] and the other two studies selected both of them as the OHRQoL questionnaires [39, 40]. The follow-ups were classified into three kinds, namely after bonding, after surgery, and debonding.

**Table 4:** Characteristics of the included studies

Study	Country	Institution	Participants (N)	type of malocclusion	Outcomes	Follow-ups after bonding	Follow-ups after surgery
Park 2015	Korea	Not reported	SFA group: 9F, 2 M CTM group: 12F, 3 M	skeletal Class III	OQLQ	–	3 months, debonding
Huang 2016	China	Department of Orthodontic at the Stomatology Hospital of Wen Zhou Medical University	SFA group: 13F, 12 M CTM group: 13F, 12 M	skeletal Class III	OHIP-14	1, 6, 12, 18 months	1, 6, 12 months, debonding
Feu 2017	Brazil	Rio de Janeiro State University	SFA group: 8 CTM group: 8	skeletal Class III	OQLQ, OHIP-14	1, 3, 6, 12, 24 months	–
Pelo 2016	Italy	Department of Surgical Sciences for Head and Neck Diseases at Catholic University of Sacred Heart	SFA group: 15 CTM group: 15	skeletal Class II/III	OQLQ, OHIP-14	–	1 month, debonding

Conventional Three-stage Method, *CTM* Surgery First Approach, *SFA* Number, *N* Female, *F* Male, *M* Orthognathic Quality of Life Questionnaire, *OQLQ* 14-item Oral Health Impact Profile, OHIP-14

### Quality assessment

According to the GRADE quality analysis, all the included studies were graded as moderate value of evidence, i.e. Grade B (Table 5).

**Table 5:** Quality assessment

Study	Study design	Study type	Definitive grade
Park 2015	Retrospective	Non-randomized controlled trial	B
Huang 2016	Prospective	Non-randomized controlled trial	B
Feu 2017	Prospective	Non-randomized controlled trial	B
Pelo 2017	Prospective	Randomized controlled trial	B

**Fig. 2:**
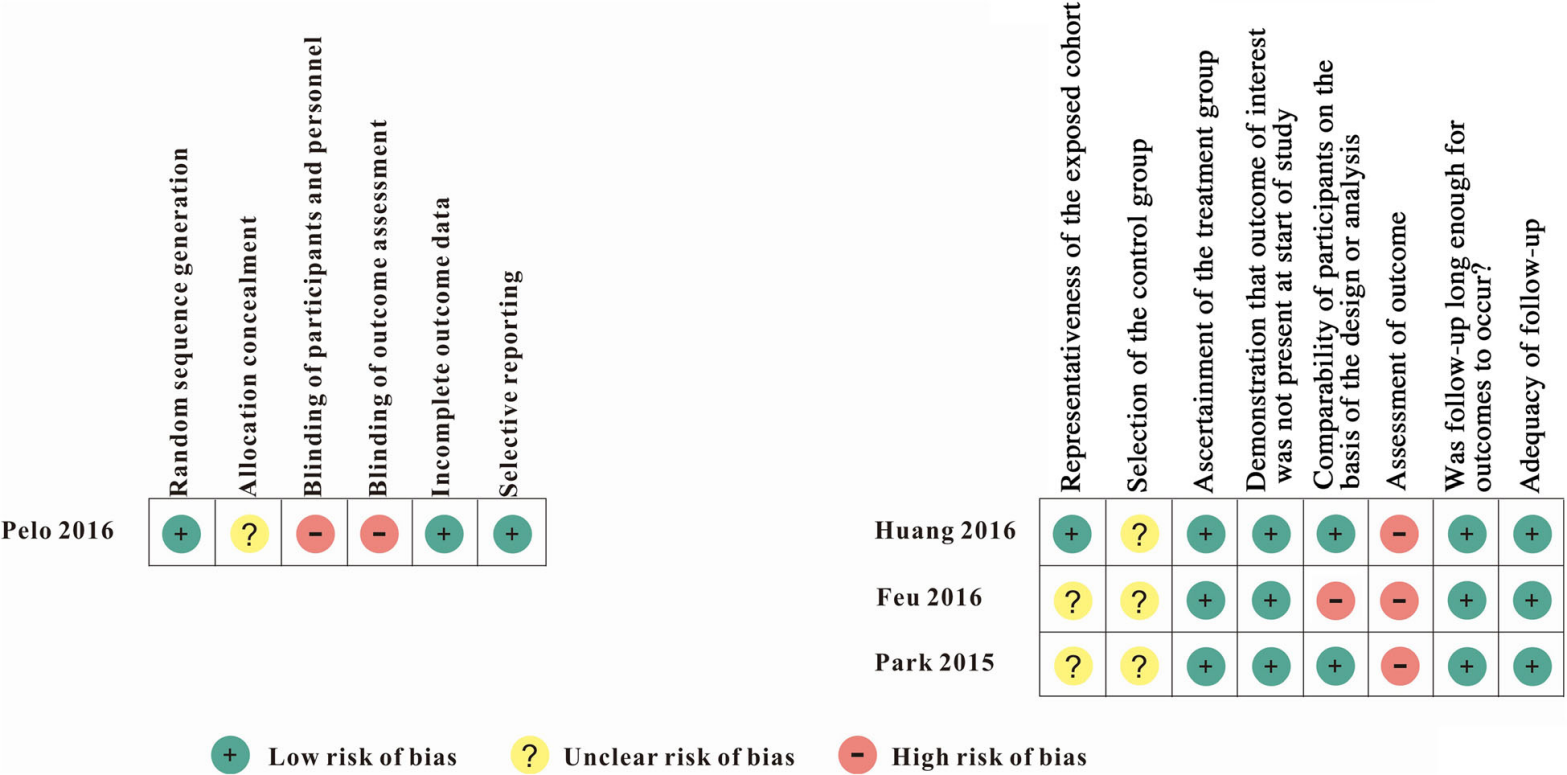
Risk of bias summary

**Fig. 3:**
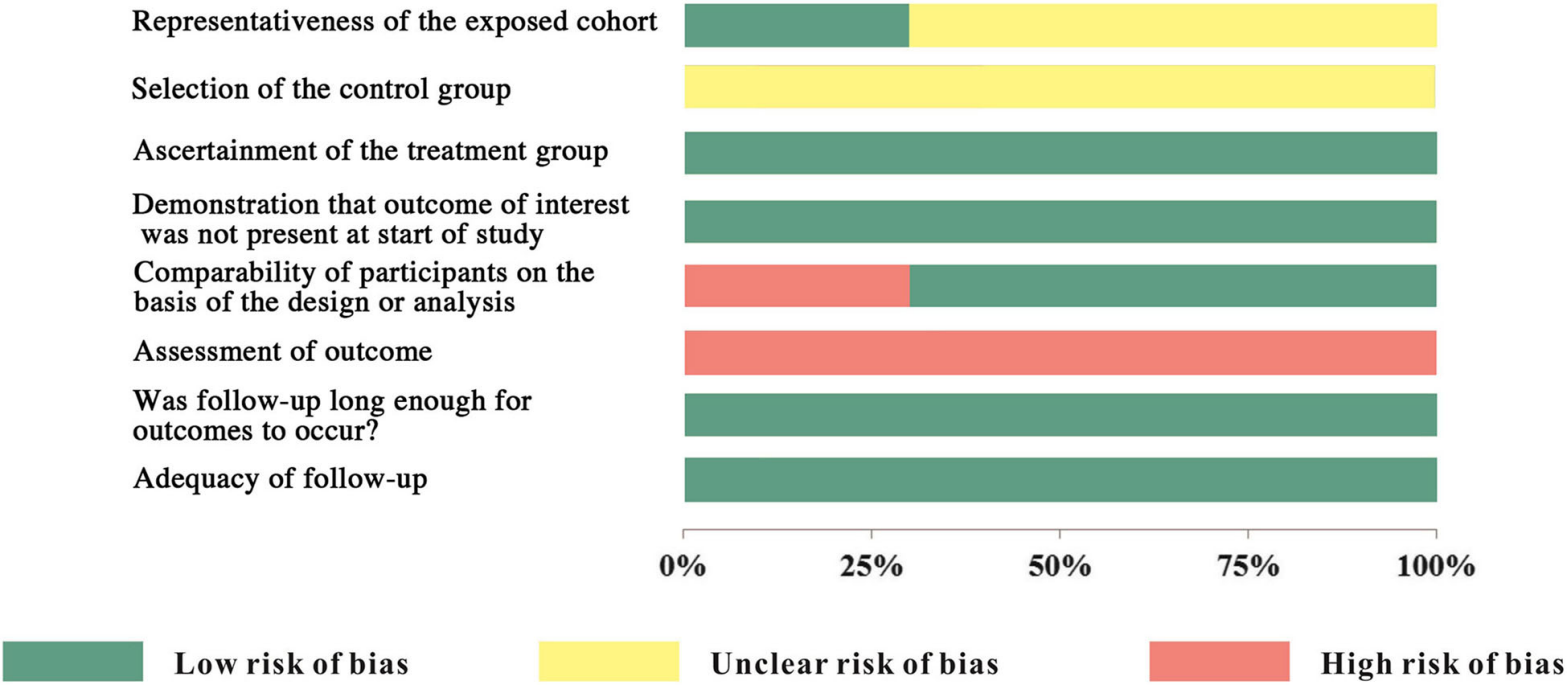
Risk of bias graph

All the included studies lacked blinding of participants and personnel as well as outcome assessment (Figs. 2, 3) [37–40]. As the orthognathic surgery and orthodontic braces were evident, it is difficult to blind patients and clinicians.

There were no incomplete outcomes data and selective reporting in the prospective randomized clinical trial by Pelo et al., but this study did not mention the method of allocation concealment [40] (Fig. 2).

As for the three non-RCTs [37–39], the ascertainment of intervention was securely recorded. Participants in treatment group were comparable to those in control group [37, 38], except for the study of Feu et al. [39]. No drop-outs were found in the included studies [37–39]. In the study by Huang et al., the selection of participants was representative in the community [38]. However, all the non-RCTs did not mention the selection of control group [37–39] (Figs. 2, 3)

### The scores of OHIP-14

In the study of Feu et al., there was significant difference between SFA group and CTM group at baseline, and two patients in SFA group accomplished surgery 3 months after their braces bonded while others in SFA group underwent surgery as soon as braces bonded [39]. These factors could result in unnegleted clinical heterogeneity, which made this study not meta-analyable. The scores of OHIP-14 in group CTM and group SFA at different time-points after bonding, which were reported by Huang et al. [38] and Feu et al. [39], were showed in Table 6.

**Table 6:** The scores of OHIP-14 in group CTM and group SFA at different time-points after bonding

	Intervention/number	Baseline	1 m	3 m	6 m	12 m	18 m	24 m
Feu 2016	SFA/8	25.4 ± 5.6	26.9 ± 10.6	17.0 ± 9.7	14.9 ± 11.0	7.5 ± 6.6	not reported	8.1 ± 5.7
	CTM/8	21.5 ± 9.0	20.4 ± 4.6	15.0 ± 6.7	11.1 ± 8.7	14.1 ± 11.3	not reported	22.1 ± 11.8
Huang 2016	SFA/25	38.68 ± 4.35	27.72 ± 3.26	not reported	13.94 ± 2.13	6.9 ± 1.39	4.11 ± 0.49	not reported
	CTM/25	39.55 ± 4.15	41.67 ± 4.14	not reported	48.48 ± 3.91	28.86 ± 3.83	15.61 ± 2.49	not reported

At 1 month after bonding, Feu et al. reported 6 participants in SFA group have underwent orthognathic surgery, and the score of OHIP-14 in CTM group was smaller than that in SFA group (*P* = 0.16) [39]. However, all of 25 participants have underwent orthognathic surgery at this time-point in Huang et al., and it is showed that the score of OHIP-14 in SFA group was smaller significantly than that in CTM group (*P* < 0.001) [38].

When bonded for 6 months, the score of OHIP-14 in CTM group kept smaller than that in SFA group without significance (*p* = 0.49) in the study by Feu et al. [39], while Huang et al. reported the results in diverse direction that the SFA group showed smaller OHIP-14 score significantly (*P* < 0.001) [38].

At 12 months after bonding, the scores of OHIP-14 in both studies (by Feu et al. and by Huang et al.) were smaller in SFA group than those in CTM group (*P* = 0.25 and *P* < 0.001, respectively) [38, 39].

**Fig. 4:**
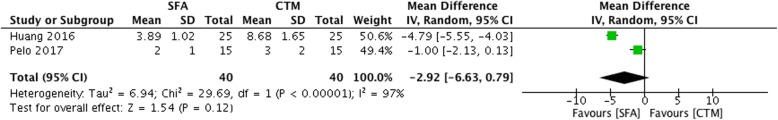
The summary score of OHIP-14 in the SFA group and the CTM group on debonding

On debonding, the scores of OHIP-14 were showed in two studies (Huang et al. and Pelo et al.) with 40 patients in SFA groups and 40 patients in CTM groups [38, 40], which were combined statistically in meta-analysis. The random effect model was selected as the heterogeneity was high (I^2^ = 97%, *P* < 0.001). The summary score of OHIP-14 in SFA group was 2.92 smaller without significance (*P* = 0.12) (Fig. 4).

### The scores of OQLQ

Over the period of 2-year follow-up after bonding, the OHRQoL of the patients in SFA group showed improvement generally. The score of OQLQ in SFA group was 6.1 at 1 year after bonding, which was much smaller than that in CTM group significantly (*P* < 0.01) [39]. When bonded for 2 years, the score of OQLQ in SFA group kept smaller than that in CTM group (*P* < 0.01) and 14.1 smaller than that at baseline (*P* < 0.01) [39].

**Fig. 5:**
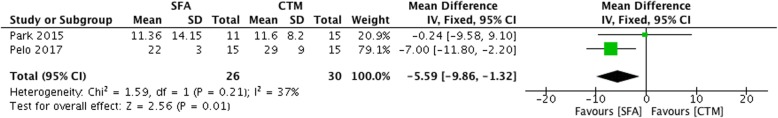
The score of OQLQ in the SFA group and the CTM group when the brackets were debonded through fixed model

When the brackets were debonded, the scores of OQLQ were reported in two studies (Park et al. and Pelo et al.) with 26 participants in the SFA groups and 30 participants in the CTM groups [38, 40], which were combined statistically in meta-analysis. The fixed effect model was selected for the low heterogeneity (I^2^ = 37%, *P* = 0.21). The summary score of OQLQ in SFA group was 5.59 smaller significantly (*P* = 0.01) (Fig. 5).

As there were only 2 studies included for each forest plot, it is hard to leave one out to explore the source of heterogeneity among studies.

## Discussion

Facial esthetics has a vital influence on personal self-confidence and interpersonal relationships, which drives the patients with dentofacial deformity to undergo orthognathic surgery [41–43]. CTM and SFA, two kinds of ortho-surgical treatments, are different in the treatment sequence, duration, patient compliance, and degree of patients’ satisfaction, which could make OHRQoL widely divergent.

This systematic review was performed to compare the effect of SFA with CTM on the OHRQoL of patients. Although SFA has been used for more than a decade, only a few studies focused on this issue. For that reason, there were only 4 suitable articles to be included [37–40].

OHIP-14, a kind of self-rating questionnaires, is invented to assess the patients’ OHRQoL. It contains 14 questions, which are divided into 7 sections, including functional limitation, physical pain, psychological discomfort, physical incapacity, psychological incapacity, social incapacity, and difficulty doing usual jobs [41]. As the reliability and validity of OHIP-14 have been tested in different countries and languages, it is utilized to describe patients’ OHRQoL widely [44–46]. It was indicated a smaller score of OHIP-14 in SFA group than that in CTM group at 1 month after bonding [38]. This result can be owing to the fact that the patients in SFA group had finished the surgery and had facial profile improved immediately at this time-point, and SFA made the patients achieve similar degree of satisfaction and dentofacial harmony to those with normal skeletal relationship [47]. At the same time-point, the patients in CTM group were just at the beginning of presurgical orthodontics and had worse facial deformity as a result of dental decompensation by CTM. Anterior crossbite was reported to influence esthetics and mastication function negatively, which contributed to a worse OHRQoL during the presurgical orthodontic stage [48, 49]. It was also reported that 73.6% patients with dentofacial deformities were uncomfortable by their appearance and almost half of them felt functional limitations before surgery, and esthetic and functional domains of QoL were improved after surgery [50]. A contrary result, however, was demonstrated in the study by Feu et al. [39]. It might be due to the differences of both groups at baseline and only 6 of the patients in SFA group who had finished surgery at 1 month after bonding.

The OQLQ, developed by Cunningham, is another self-rating questionnaire invented to assess the patients’ OHRQoL, and is also validated all around the world, especially in measuring the influence of dentofacial deformities and the benefits of orthognathic surgical treatment on patients’ OHRQoL [51–53]. Over the period of 2-year follow-up after bonding, the OHRQoL of the patients in SFA group, which was measured by OQLQ, showed improvement generally and kept better than that in CTM group [39]. After debonding, the summary scores of OHIP-14 and OQLQ of the patients treated with SFA were smaller than CTM. These results might be contributed by side effects of the presurgical orthodontics of CTM such as white spot and periodontal inflammation [54, 55], and the shorter treatment duration as well as the less complication of SFA [56].

It is necessary for doctors, however, to control the inclusion criteria of SFA strictly. Previous studies enrolled patients with skeletal Class III malocclusion with a flat curve of Spee who were randomized to two independent groups, while a number of patients with these characteristics were not identified by doctors [22]. In spite of the accelerating phenomenon in SFA, there were still several patients failing to finish the whole treatment in 2 years [39]. It is reported that patients treated by SFA had lower compliance at the metaphase of treatment, which might be due to the fact that their worst trouble, the facial deformity, had been solved by the surgery at the beginning, while braces and other devices such as micro-implant anchorage became an annoyance in their lives during the treatment [57]. Therefore, the treatment duration of SFA may not match the expected results.

There were some limitations in our systematic review and meta-analysis. Four studies were included in this systematic review, and totally three of these studies were included in the meta-analysis. However, only two studies were included in each forest plot, which might bias the summary results. In addition, there was only one RCT included eventually. Therefore, multi-center prospective randomized clinical trials with large sample size are needed to confirm the results in our systematic review and meta-analysis. Furthermore, the studies included did not analyse the impact of gender, socioeconomic factors, and individual behavior on the OHRQoL of the patients treated with SFA or CTM, which remains to be researched in further studies.

## Conclusion

This systematic review and meta-analysis showed that current evidence supported the better OHRQoL outcomes of patients treated by SFA than CTM. However, there was only one RCT included eventually. The limited numbers of enrolled participants tent to increase the bias as well. Large sample and well-designed RCTs are needed to validate and confirm the results in this meta-analysis in future.

## Data Availability

The summary of data extraction in this study is available upon request to the corresponding author.

## Abbreviations

CCTs: Controlled clinical trials
CTM: Conventional three-stage method
OHIP-14: 14-item Oral Health Impact Profile
OHRQoL: Oral health-related quality of life
OQLQ: Orthognathic Quality of Life Questionnaire
RCTs: Randomized controlled trials
SFA: Surgery first approach
